# PUBLIC BATH USE AT A JAPANESE SENIOR APARTMENT ASSOCIATED WITH NEIGHBORHOOD RELATIONSHIPS AND SOCIAL SUPPORT

**DOI:** 10.1093/geroni/igad104.2728

**Published:** 2023-12-21

**Authors:** Mai Takase, Isuzu Nakamoto, Hiroshi Murayama

**Affiliations:** Tokyo Metropolitan Institute for Geriatrics and Gerontology, Tokyo, Tokyo, Japan; Health Sciences, Graduate School of Medicine, Tohoku University, Aoba-ku, Sendai, Miyagi, Japan; Tokyo Metropolitan Institute of Gerontology, Tokyo, Tokyo, Japan

## Abstract

The number of older adults who choose to move into senior apartments is increasing in Japan. The development of quality relationships within the senior apartment is important for the healthy aging of the residents. A “public bath” is a unique facility in Japanese senior apartment where residents can interact with each other. Historically, Japan has had a culture of bathing with family and friends in public baths, and the use of this facility is expected to lead to socialization. This study aimed to examine the association between the frequency of public bath use, and neighborhood relationship and social support within a senior apartment. A cross-sectional survey was conducted in a senior apartment in February 2022 (Saitama Prefecture, Japan) towards the entire residents (n=185). Frequency of public bath use (once a week or more vs. less than once a week) was used as the independent variable. Neighborhood relationship (can support or talk to each other vs. only light greetings or no relationship) and social support (have vs. do not have social support) within the senior apartment were used as dependent variable. A binary regression analysis (n=117, female=60.7%) revealed that a higher frequency of public bath use associated with deep neighborhood relationship (OR: 7.14, 95% CI: 1.39-36.63) and social support (OR: 3.44, 95% CI: 1.19-9.99). These results suggest the importance of having a public bath in Japanese senior apartment setting. Furthermore, integrating facilities that possess cultural significance at senior apartments could be important for fostering social relationships.

